# Orthology prediction methods: A quality assessment using curated protein families

**DOI:** 10.1002/bies.201100062

**Published:** 2011-10

**Authors:** Kalliopi Trachana, Tomas A Larsson, Sean Powell, Wei-Hua Chen, Tobias Doerks, Jean Muller, Peer Bork

**Affiliations:** 1) Structural and Computational Biology Unit, European Molecular Biology LaboratoryHeidelberg, Germany; 2) Developmental Biology Unit, European Molecular Biology LaboratoryHeidelberg, Germany; 3) Institute of Genetics and Molecular and Cellular Biology, CNRS, INSERM, University of StrasbourgStrasbourg, France; 4) Genetic Diagnostics Laboratory, CHU Strasbourg Nouvel Hôpital CivilStrasbourg, France; 5) Max-Delbruck-Centre for Molecular MedicineBerlin-Buch, Germany

**Keywords:** metazoan, orthology, quality assessment

## Abstract

The increasing number of sequenced genomes has prompted the development of several automated orthology prediction methods. Tests to evaluate the accuracy of predictions and to explore biases caused by biological and technical factors are therefore required. We used 70 manually curated families to analyze the performance of five public methods in Metazoa. We analyzed the strengths and weaknesses of the methods and quantified the impact of biological and technical challenges. From the latter part of the analysis, genome annotation emerged as the largest single influencer, affecting up to 30% of the performance. Generally, most methods did well in assigning orthologous group but they failed to assign the exact number of genes for half of the groups. The publicly available benchmark set (http://eggnog.embl.de/orthobench/) should facilitate the improvement of current orthology assignment protocols, which is of utmost importance for many fields of biology and should be tackled by a broad scientific community.

## Introduction

The analysis of fully sequenced genomes offers valuable insights into the function and evolution of biological systems 1. The annotation of newly sequenced genomes, comparative and functional genomics, and phylogenomics depend on reliable descriptions of the evolutionary relationships of protein families. All the members within a protein family are homologous and can be further separated into orthologs, which are genes derived through speciation from a single ancestral sequence, and paralogs, which are genes resulting from duplication events before and after speciation (out- and in-paralogy, respectively) 2, 3. The large number of fully sequenced genomes and the fundamental role of orthology in modern biology have led to the development of a plethora of methods (e.g. 4–11) that automatically predict orthologs among organisms. Current approaches of orthology assignment can be classified into (i) graph-based methods, which cluster orthologs based on sequence similarity of proteins, and (ii) tree-based methods, which not only cluster, but also reconcile the protein family tree with a species tree (Box 1). Despite the fact that orthology and paralogy are ideally illustrated through a phylogenetic tree, where all pairwise relationships are evident, tree-based methods are computationally expensive and at times fail due to the complexity of the family or to the substantial number of species in the comparison 12. As a trade-off between speed and accuracy, the evolutionary relationships among proteins in comparisons that include a large number of species are better explored using graph-based methods. During the first large-scale orthology assignment project of multiple species, the concept of clusters of orthologous groups (COGs) was introduced 4. A COG consists of proteins that have evolved from a single ancestral sequence existing in the last common ancestor (LCA) of the species that are being compared, through a series of speciation and duplication events 4. The orthologous/paralogous relationships among proteins of multiple species are better resolved through orthologous groups (OGs) rather than pairs of orthologs. This is particularly evident in the instances of complex protein family histories (e.g. tubulins) or families over significant phylogenetic distances (e.g. proteins conserved across all domains of life) 13.

**Box 1 Comparison of orthology prediction methods**

Orthology prediction methods can be classified based on the methodology they use to infer orthology into (i) graph-based and (ii) tree-based methods 12, 16, 17. Different graph-based methods are designed to assign orthology for two (pairwise) or more (multiple) species. Graph-based methods assign proteins into OGs based on their similarity scores, while tree-based methods infer orthology through tree reconciliation.

**Pairwise species methods (e.g. BHR, InParanoid, RoundUp):**

Based on these methods, orthologs are best bi-directional hits (BBH) between a pair of species. BRH 46 is the first automated method and does not detect paralogs. InParanoid 47 implements an additional step for the detection of paralogs. RoundUp 48 uses evolutionary distances instead of BBH. In addition to the restriction of only two-species at a time, these methods are disadvantageous for long evolutionary distances.

**Multi-species graph-based methods (e.g. COG, eggNOG, OrthoDB, OrthoMCL, OMA):**

Due to the fast implementation and high scalability, there are many graph-based methods for multi-species comparisons. So far, all of them use either BLAST or Smith-Waterman (e.g. PARALIGN, SIMG) as sequence-similarity search algorithms. However, they are quite diverse regarding the clustering algorithms. COG, eggNOG, and OrthoDB share the same methodology: they identify three-way BBHs in three different species and then merge triangles that share a common side. OrthoMCL is a probabilistic method that uses a Markov clustering procedure to cluster BBH into OGs. OMA removes from the initial graph BBHs characterized by high evolutionary distance; a concept similar to RoundUp. After that, it performs clustering based on maximum weight cliques. Unique database characteristics are the hierarchical groups (OGs in different taxonomic levels) and “pure orthologs” (generate groups of one-to-one orthologs without paralogs), which has been introduced only by OMA (indicated as ** in the figure). Hierarchical groups can substitute the view of phylogenetic trees.

**Multi-species tree-based methods (e.g. TreeFam, Ensembl Compara, PhylomeDB, LOFT):**

Tree-based prediction methods can be separated into approaches that do (like EnsemblCompara, TreeFam, and PhylomeDB) and do not, e.g. LOFT 49, use tree-reconciliation. Tree-based methods also initially use homology searches; however, their criteria are more relaxed, as the orthology is resolved through tree topology. Although a reconciled phylogenetic tree is the most appropriate illustration of orthology/paralogy assignment, there are a few caveats to such an approach, namely their scalability and sensitivity to data quality.

For a more detailed and extensive discussion of the differences among orthology methodology, we recommend refs. 12, 16, 17.

Phylogenetic distribution describes the species range of each database. Homology search shows a few technical differences for recruiting orthologs. §: Supplies OGs whose members share only orthologous relationships. *: The user can compare any two genomes spanning a phylogenetic distance from bacteria to animals.

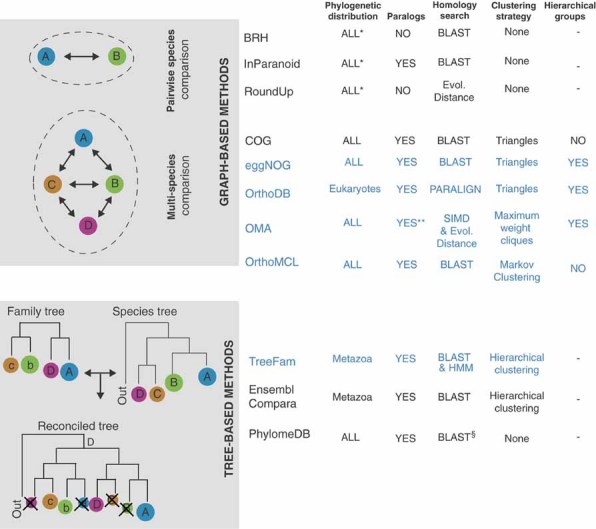

**Figure 1:**
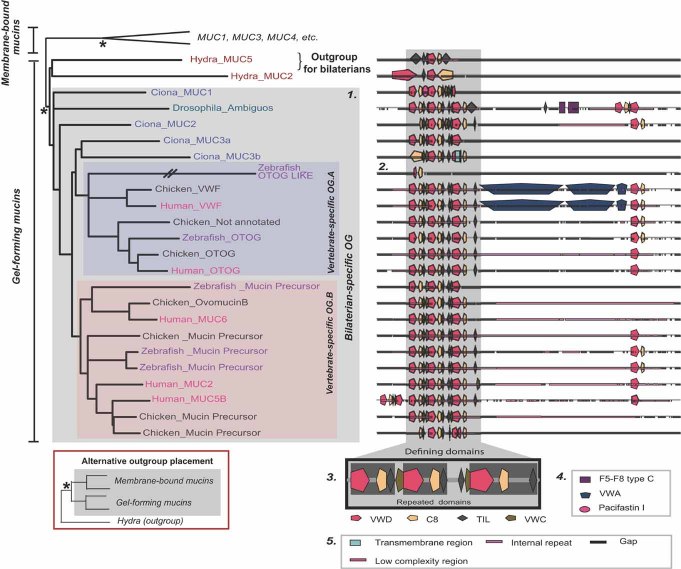
Mucins: a challenging family for orthology prediction. This figure shows the phylogenetic tree and domain architecture of aligned mucins. The identification of cnidarian (an outgroup for bilaterians) mucin2/5 orthologs separates the gel-forming mucins from other mucins, defining a bilaterian-specific OG (gray box). An alternative topology of Hydra in respect to the LCA of bilaterian species (shown schematically in the red box) would propose that those two different classes of mucins should be clustered together at the bilaterian level. The bilaterian OG can be further resolved at the vertebrate level into OG.A (blue) and OG.B (red), illustrating the hierarchical nature of OGs. This family, besides its large size due to vertebrate-specific duplications, exemplify five additional problems that often lead to orthology misassignment: (1) uneven evolutionary rate illustrated as branch lengths, lowering the sequence similarity among members of the family; (2) quality of genome annotation: the particular zebrafish protein can be either a derived member of the mucin family or a erroneous gene prediction; (3) repeated domains: the domain combination VWD-C8-VWC, which is the core of the family, is repeated multiple times within the protein; (4) complexity of domain architectures: there are multiple unique domain combinations (e.g. the VWD domain is combined with the F5-F8 type C domain only in the *Drosophila* ortholog); and (5) low complexity regions: internal repeats within the amino acid sequences and other low complexity features impede the correct sequence alignment of the mucins. *Possible orthologous sequence at the LCA of cnidarians bilaterians.

Despite the clear definition of OGs, their automated prediction is challenged by a number of biological and technical factors exemplified by the evolution of mucins (see Fig. 1), a family with a complex evolutionary history 14. The phylogenetic tree of mucins resolves the orthologous relationships among the members of the family at every evolutionary level (Fig. 1). Still, how they are grouped into OGs depends on the phylogenetic range of the species compared. For instance, a vertebrate-specific OG will include otogelin and VWF mucins, but not the additional gel-forming mucins (MUC5, MUC2, and MUC6). Conversely, all gel-forming mucins encompass a large OG when considering bilaterians (an animal clade that includes vertebrates, insects, and nematodes among others) as the level of comparison. Analyzing the OGs at different taxonomic levels (e.g. vertebrates vs. bilaterians) sheds light on the evolutionary history of the family; however, big protein families, which have expanded and contracted many times in the history of a lineage, require an increased resolution of orthologous-paralogous relationships within the same taxonomic level. The inclusion of outgroup species of a taxonomic level delineates the aforementioned relationships. For instance, Hydra sequences revealed the existence of two paralogous sequences in the LCA of bilaterians (marked by an asterisk in Fig. 1); thus, according to the OG definition, membrane-bound and gel-forming mucins should be clustered into two different OGs. Despite the lineage-specific duplications and losses of domains 14, many complex domain architectures are found across the family but not always conserved, which contributes to erroneous assignments of orthologs. Repeated domains and fast-evolving mucin domains also hamper the automatic sequence alignment of the family 15. All these factors and more (see Fig. 1) can influence the accuracy of the many emerging resources for orthology assignment 13, 16, 17. To understand the impact on individual resources, one needs to understand the design of different orthology prediction methods (briefly introduced in Box 1). However, an appropriate comparison is extremely difficult for two major reasons, both of which contribute to conflicting orthology assignments: (i) each method differs in technical (e.g. species distribution, similarity cut-offs) and conceptual (e.g. OG definition) aspects, and (ii) the lack of a common set of species obtained from the same release of genome repositories and tested across all methods 16.

## Benchmarking orthology prediction methods using a phylogeny approach

Despite the acknowledged necessity of a phylogeny-based evaluation of orthology, thus far the majority of quality assessment tests are based on the functional conservation of predicted orthologs 18–21. However, orthology is an evolutionary term defined by the relationships among the sequences under study, and functional equivalences are not always inferable 13. Moreover, the functional divergence between orthologs and paralogs (sub-/neo-functionalization of paralogs) or alteration of function during long evolutionary distances 13 suggests that those tests are biased toward single copy genes or conserved families and less suited for large diversified families. It has been proposed that the inclusion of synteny information limits the errors arisen due to low sequence similarity and increases orthology accuracy 22. However, this requires a certain level of synteny conservation among the compared species. It has been illustrated that synteny information combined with sequence similarity identifies accurately the paralogs that have arisen through WGD in six closely related yeast species 23. Further refinement of this dataset using tree reconciliation 24, 25 ends up with a phylogeny-based dataset. However, it is still biased toward simple evolutionary scenarios, highlighting mostly the impact of lineage-specific losses in orthology prediction 26. For a much more fine-grained analysis that also involves complex OGs, we developed a phylogeny-based benchmark set and applied it to a much more diverse taxonomic clade, namely metazoans. The set involved the manual curation of the phylogeny of 70 protein families that range from single copy orthologs to OGs with 100 members (Table S1 of Supporting Information). The phylogenetic analysis of each protein family for 12 reference bilaterian species and 4 basal metazoans as outgroups (Box 2) resulted in the reference orthologous groups (RefOGs), including in total 1,638 proteins.

**Box 2 Phylogenetic analysis of the 70 protein families**

Selecting families for exploring caveats of orthology prediction: we focused on five major affecting factors of orthology prediction, mostly related with metazoan (eukaryotic) biology: rate of evolution (fast- vs. slow-evolving families), domain architecture (single domain vs. multiple repeated domains), low complexity/repeats, lineage-specific loss/duplication (single copy families vs. multiple duplication events), and alignment quality (high- vs. low-quality alignment). We used the eggNOG database to select 70 families (Supporting Information) that we refer to as “homology seeds.” Of the selected families, 35 exemplify known biological and technical challenges. Five additional slow-evolving, well-aligned families were chosen as counterbalance, while the remaining 30 families were chosen randomly to avoid prior biases (Table S1 of Supporting Information).

Defining of reference species: for an applicable comparison of the five databases studied, we had to confine the analysis to 12 reference species that are shared by all resources: *Caenorhabditis elegans*, *Drosophila melanogaster*, *Ciona intestinalis*, *Danio rerio*, *Tetraodon nigroviridis*, *Gallus gallus*, *Monodelphis domestica*, *Mus musculus*, *Rattus norvegicus*, *Canis familiaris*, *Pan troglodytes*, *Homo sapiens*. All 12 species belong to the bilaterians, a metazoan subgroup simplifies the objective of this study since (i) the phylogeny of bilaterians is reasonably defined, and (ii) a few fully sequenced basal metazoan genomes (like cnidarians) can be used as outgroups of bilaterians 29, 50–52.

The phylogenetic analysis: briefly, we selected 70 aforementioned COG/KOGs, as they exist in eggNOG v2 6, which we refer to as “homology seeds.” To exclude errors due to old genome annotation (eggNOG v2 is based on Ensembl v46), we mapped the “homology seed” identifiers to Ensembl v60. The following steps were performed uniformly to all families certifying that RefOGs are not biased toward their initial “homology seeds.” BLAST 53 searches were performed in the 16 animals using query sequences from well-annotated genomes (e.g. human, zebrafish, and fly). The homologous sequences were aligned with MUSCLE 54 and the alignments were used to build initial NJ trees with Clustal X 55 (indicated as Round 1 in the illustration below). Large groups were thereafter divided based on the positions of orthologs in the outgroups, as exemplified by the family of mucins (Fig. 1). In several cases where no clear outgroup was found, RefOGs were defined based on (i) the domain content, (ii) manual inspection of the alignments, and (iii) previous published descriptions of the families. After the initial curation of the families, all sequences determined to be members of the bilaterian RefOGs were aligned using MUSCLE 54. Alignments were refined 56 and hidden Markov models (HMM) were built using the HMMER3 package 57. In a second refinement step (indicated as Round 2), the HMM models were used to identify related sequences that were left out from the 16 aforementioned genomes. As a last step, all qualified members of each RefOG were realigned, using the same procedure as before, final HMM models were generated and phylogenetic trees were calculated using PhyML version 3.0 58. The detailed analysis is described in the supplementary file. Black arrows indicate the flow of the analysis. * Steps that are repeated after HMM profile searches resulting in RefOGs after Round 2 (red arrow).

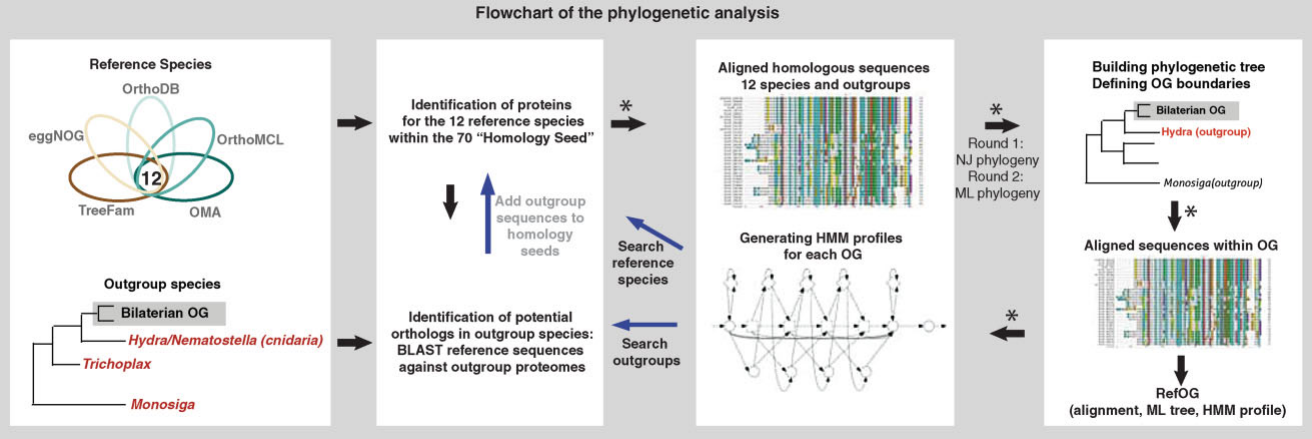

The manually curated benchmarking set was used for two different analyses: (i) comparison of RefOGs to the automatically predicted OGs of five publicly available databases, and (ii) comparison of RefOGs to different customized versions of the eggNOG database. The first comparison aimed at demonstrating the power of this dataset to guide the improvement of current methods. We selected five databases, namely TreeFam 5, eggNOG 6, OrthoDB 7, OrthoMCL 8, and OMA 9, since each is designed for multiple-species comparison, but with unique database features (Box 1). Although the comparisons are against the same benchmarking set, we are aware of several other confounding variables, such as algorithmic differences, species representation/distribution or genome annotation, that can all affect the results. Yet, it quantifies the status of the compared databases in an objective way. To quantify the impact of some specific biological and technical factors, we additionally generated different versions of the eggNOG database to monitor several influencing factors one by one.

**Figure 2:**
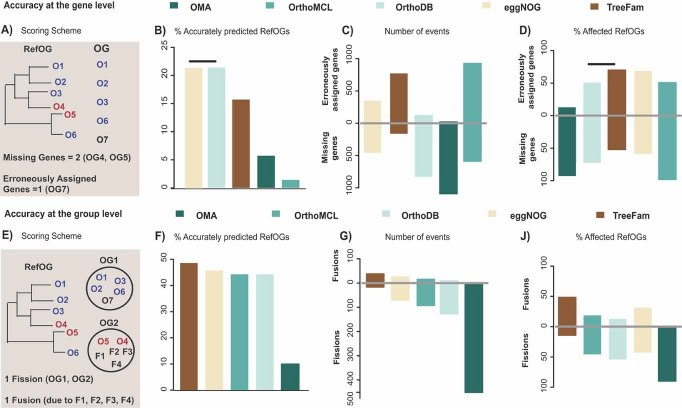
The 70 manually curated RefOGs as a quality assessment tool. Five databases were used to illustrate the validating power of the benchmark set. The performance of each database was evaluated at two levels: gene (focus on mispredicted genes; upper panel) and Group (focus on fusions/fissions; lower panel) level. **A:** Gene count – for each database we identified the OG with the largest overlap with each RefOG and calculated how many genes were not predicted in the OG (missing genes) and how many genes were over-predicted in the OG (erroneously assigned genes) and **E:** group count – for each method we counted the number of OGs that members of the same RefOG have been separated (RefOG fission) and how many of those OGs include more than three erroneously assigned genes (RefOG fusion). To increase the resolution of our comparison, three different measurements for each level were provided, resulting in six different scoring schemes. **B:** Percentage of accurately predicted RefOGs in gene level (RefOGs with no mispredicted genes); **C:** number of erroneously assigned and missing genes; **D:** percentage of affected RefOGs by erroneously assigned and missing genes; **F:** percentage of accurately predicted RefOGs in grouplevel (all RefOG members belong to one OG and are not fused with any proteins); **G:** number of fusions and fissions; and **J:** percentage of affected RefOGs by fusion and fission events. Databases are aligned from the more to the less accurate, taking into account the total number of errors (length of the bar in total). Black bars indicate identical scores.

We assessed the quality of the OGs at two different levels of resolution: (i) gene count, measuring mispredicted genes, and (ii) group count, reflecting errors at the level of OG (Fig. 2). Additionally, for each of the two resolution levels, we used three counting schemes allowing us to distinguish database-specific trends. At a strict requirement of all genes being correctly assigned (gene count level) only as little as 3–22% of the RefOGs were recovered, while a more relaxed requirement that curated orthologs are not clustered in multiple OGs or with other homologous proteins that are not part of the RefOG (group count level) results in 10–48% correctly predicted RefOGs. Limiting our analysis to the 35 most challenging families decreases this percentage even more (Fig. S1 of Supporting Information), reflecting our initial aim to select families that hamper accurate orthology prediction; we aimed at a benchmark set that points out shortcomings of each method and leads to its improvement. All above indicated that there is room for improvement for all methods, but most importantly, we have to understand which factors contributed to this result.

## The phylogenetic range of the compared species affects the accuracy of prediction

The phylogenetic distribution of the compared species influences the orthology/paralogy assignment, as we exemplified with the mucin family (vertebrate- vs. bilaterian-specific groups). The broader the phylogenetic range of the compared species the larger the OGs, as the single ancestral sequence from which all the orthologs and paralogs are derived is placed deeper in the tree. This is reflected in the ranking of the five databases that varies considerably in the six different scoring schemes used (Fig. 2). For instance, although OrthoMCL contains the highest number of erroneously assigned genes (Fig. 2C), the number of RefOGs that are affected by erroneously assigned genes is higher for eggNOG than OrthoMCL (Fig. 2D). On closer examination, OrthoMCL overpredicts many orthologs in only a few families, while eggNOG overpredicts a few proteins in many families (Table S2 of Supporting Information), partially due to mispredicted genes (later characterized as pseudogenes or wrong gene models) inherited from an old genome annotation (see below). We assume this observation is partly due to the diverse species ranges of the studied repositories (Box 1). EggNOG, although it provides a broad species coverage (630 prokaryotes and 55 eukaryotes), supplies OGs for several taxonomic levels, such as metazoans (meNOGs) that are used in this study and are build from 34 bilaterians in the eggNOG version studied here. On the other hand, OrthoMCL builds its OGs from all 138 eukaryotic and prokaryotic species in the database. In other words, ancient families, e.g. ABC transporters, which expanded before the bilaterian radiation, form huge OGs in OrthoMCL, but not in the meNOG subset of eggNOG. As different scientific questions require a different species range, hierarchical groups as provided by eggNOG 27, OrthoDB 28, and OMA 9 appear to be a balanced solution to serve many different questions, compared to databases that are only dedicated to a particular phylogenetic range [be they narrow (TreeFam) or broad (OrthMCL)].

Despite being specifically designed for metazoans, TreeFam has the second largest number of erroneously assigned genes after OrthoMCL (Fig. 2C), which is accompanied by the largest number of fusion events (Fig. 2G). This can be attributed to the choice of outgroups used by Treefam. TreeFam families are phylogenetically separated by a non-animal outgroup (yeast or plant), while, for example, *Monosiga brevicollis* 29 or other proposed species 30 would be much better suited. The choice of a phylogenetically closer species would presumably split artificially large families. Furthermore, delineating orthology through tree reconciliation benefits TreeFam in the category of missing genes (Fig. 2C), since the lack of a closer outgroup prevents the bilaterian OGs from splitting, as illustrated in Fig. 1. In contrast, the database with the largest number of missing genes and fission events is OMA (Figs. 2C and G) due to an alternative operational definition of an OG 31; only proteins with one-to-one orthologous relationships are included in an OG, so that large families with multiple paralogs are split artificially into multiple smaller OGs. The latest release of the OMA database, publicly available after the completion of our analysis, has been redesigned and now provides OGs based on both OMA and COG formulations 9.

In summary, the initial design of an orthology resource, e.g. phylogenetic range of species, “hierarchical groups”, or formulation of OG, is crucial for its performance. In any case, all methods only predict a fraction of RefOGs accurately and mispredict a large number of genes (Fig. 2). It is noteworthy that there are RefOGs that none of the methods infer accurately, indicating that there are biological and technical factors that affect the performance of orthology assignment more generally. We have thus tried to relate a few of them with the outcome of this comparison.

## The impact of family complexity on orthology prediction

**Figure 3:**
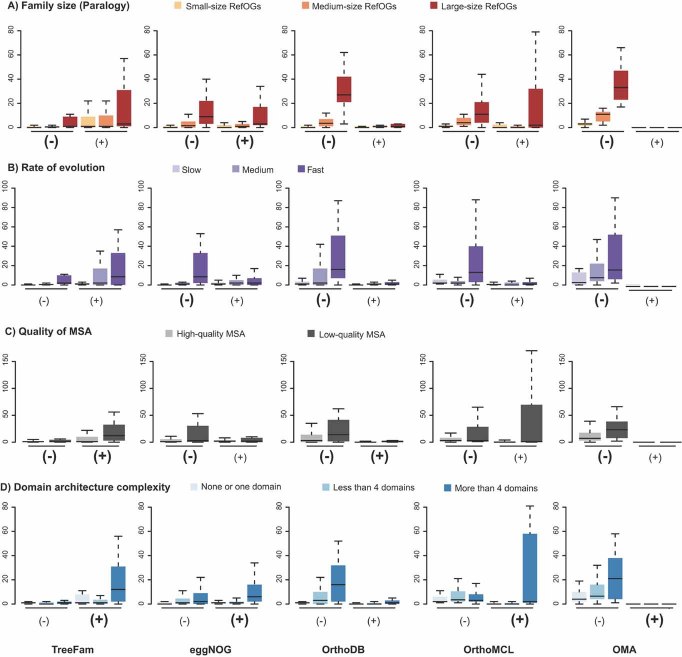
The impact of biological complexity in orthology assignment. To evaluate the impact of important caveats in orthology prediction, the RefOGs were classified based on their family size, rate of evolution, alignment quality and domain complexity. **A:** Family size (reveals the impact of paralogy): the RefOGs were separated into (i) small (less than 14 members), (ii) medium (more than 14 members, but less than 40), and (iii) large (more than 40 genes). **B:** Rate of evolution: the RefOGs were classified based on the MeanID score (described as the “FamID” in 33), an evolutionary rate score derived from the MSA of each family. There are: (i) slow-evolving (MeanID >0.7), (ii) medium-evolving (MeanID <0.7, but >0.5), and (iii) fast-evolving (MeanID <0.5) RefOGs. **C:** Quality of alignment: we classified the families based on their norMD score 34 into: (i) high-quality alignment (norMD >0.6), and (ii) low-quality alignment 44, 45. We can observe that high amino acid divergence correlates with an increasing number of mispredicted genes. **D:** Domain architecture complexity; each RefOG is associated with the average number of domains, which is equal to the sum of predicted domains of the members of one RefOG divided by the family size. There are three levels of complexity, starting from (i) none or one domain on average, to (ii) two to four, to (iii) more than four. We observe that the performance of the five databases correlates with the biological complexity of RefOGs; as families increasing their complexity (more members, fast-evolving or multiple domains), the accuracy of predictions drops. (+) and (−) symbolize erroneously assigned and missing genes, respectively. Significant correlations (Table S5 of Supporting Information) between the distribution of missing/erroneously assigned genes and the tested factor are indicated in bold [**(+)**, **(−)**]. Figures S2 and S4 of Supporting Information show similar observations at the group level (fusions/fissions of RefOGs).

Due to the central role of orthology in comparative and functional genomics, there is an extensive literature on accuracy-restricting factors of its assignment 13, 16, 17. We have already mentioned several caveats of orthology prediction using the mucin family, the majority of which are exemplified by the 70 RefOGs. The families were selected under certain criteria (Box 2), mostly with a view to understanding the impact of a few biological and technical factors, namely duplications (paralogy)/losses, rate of evolution, domain architecture, and alignment quality. All these factors have been reported to affect the quality of orthology prediction 17. Paralogy as manifested in multi-gene families hamper the accurate orthology prediction 4, 13. Multiple lineage-specific gene losses and duplications result in complex evolutionary scenarios, which are hard to interpret. Classifying the RefOGs based on their size, we observed that the larger the RefOG, the more mispredictions are introduced by the methods (Fig. 3A). For all methods, the numbers of missing genes (Fig. 3A) and OG fissions (Fig. S2 in Supporting Information) increases significantly with the RefOG size (Table S5 of Supporting Information). Additionally, families with more than 40 members accumulate both fusion and fission events. For instance, GH18-chitinases, a RefOG that consists of 45 members, is characterized by multiple vertebrate-specific duplication events. All graph-based methods split the vertebrate subfamilies of the GH18-chitinases into distinct groups (Table S2 of Supporting Information), and TreeFam lumps the RefOG with insect-specific homologs due to the presence of the glyco-hydro-18 domain, although phylogenetic analysis of the family indicates a general lack of orthology among those groups 32.

Some large-size RefOGs, like ribosomal proteins or SAM-synthetases are, however, predicted accurately by several methods. Since these two well-predicted large families are well conserved, we decided to investigate the impact of the rate of evolution on orthology prediction. We categorized our benchmarking families into fast-, medium-, and slow-evolving based on their MeanID score (described as the “FamID” in 33), which indicates the rate of evolution (Supporting Information). Fast-evolving families tend to accumulate a larger number of errors (Fig. 3B). All graph-based methods miss a larger number of genes and introduce more fission events (Fig. S2 in Supporting Information) in fast-evolving RefOGs compared to the more slowly evolving groups. Since the MeanID score is calculated based on the multiple sequence alignment (MSA), we investigated the impact of MSA quality by calculating the norMD score 34, an alignment score that depends on the number and the length of aligned sequences as well as their estimated similarity (Supporting Information). We expected TreeFam to be more sensitive to low-quality MSAs compared to graph-based methods, since it uses MSA for tree-building and reconciliation steps to infer orthology. Indeed, it presents the highest deviation for all sources of errors (Table S5 of Supporting Information). We also found that the number of missing genes is also affected by the alignment quality in graph-based methods (Fig. 3C). Because MeanID and norMD scores are correlated, many of the fast-evolving families are also poorly aligned. Still, we can see that TreeFam is significantly more affected by MSA quality rather than rate of evolution.

The vast majority of proteins contain only one domain, and the most common multi-domain proteins tend to have few (two or three) domains 35, 36. Due to a variety of genetic processes (duplication, inversion, recombination, retrotransposition, etc.) proteins consisting of multiple domains with independent evolutionary origin can arise 37–40. This leads to conceptual but also practical challenges (e.g. alignment) in orthology prediction, as the domains have followed distinct evolutionary trajectories 16. We identified the domains of each protein in each RefOG through the SMART database 41. Out of the 70 RefOGs, 75% contain multi-domain (more than two domains) proteins, compared to 62% in the random subset and a report of 40% multi-domain occurrence in metazoans 36, which illustrates the tendency of the benchmark set toward more challenging families. As expected, the proportion of accurately predicted RefOGs decreases as the number of average domains per family increases (Fig. 3D). Interestingly, the rate of erroneously assigned genes presents the most significant correlation with domain complexity, suggesting that protein families with multiple protein domains “attract” non-orthologous proteins due to domain sharing. Repeated domains within proteins, as the Von Willebrand factor (VW) D-C8-VWC repeat in mucins (Fig. 1) or the epidermal growth factor (EGF) domains in collagen, also lead to lower quality of OGs. All of the 27 RefOGs containing repeated domains are more error prone than RefOGs without repeated domains (Fig. S3 of Supporting Information).

Taken together, classification of the families from slow-evolving single copy to fast-evolving large families revealed method-specific limitations, but also that all pipelines fail to predict complex families accurately. The rates of missing genes and fissions significantly correlate with the family size and rate of evolution, as expected, whereas the domain complexity seems to affect the recruitment of non-orthologous genes (Fig. 3, Figs. S2 and S4 of Supporting Information).

## Species coverage affects orthology prediction

**Figure 4:**
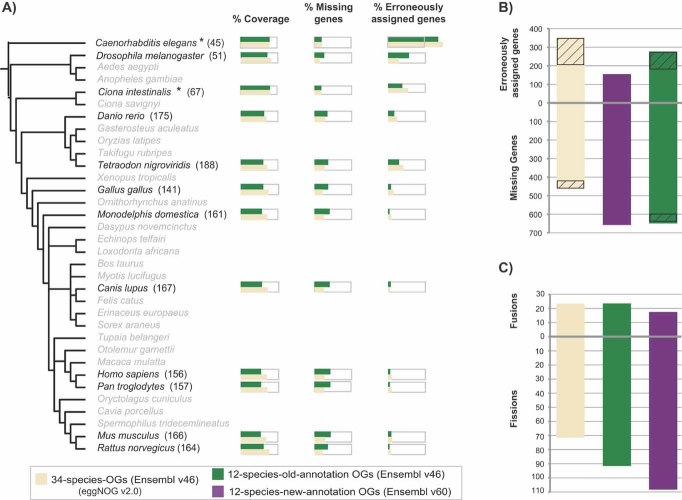
The impact of species coverage and genome annotation. **A:** Comparison of the performance of 34-species and 12-species OGs using RefOGs. We measure the percentage of orthologs recovered (coverage), missing genes and erroneously assigned genes for each reference species for those datasets [yellow bar: publicly available OGs in eggNOG (same measurements as Fig. 2) and green bar: customized OGs of the 12 selected species using same genome annotations as the public eggNOG]. The reference species are highlighted by black letters, while the unconsidered species that complete the set of 34 eggNOG species are written in gray letters. Numbers in parentheses show the total number of orthologs per species in the benchmarking set. The gray boxes enclosing the colored bars correspond to 100% coverage. Notice that the coverage is always higher for the 34-species OGs compared to the 12-species OGs except in the cases of *C. elegans* and *Ciona* (marked by asterisk), which are separated by long branches in both datasets. **B:** Comparison of the public eggNOG (yellow bar), 12-species-old-annotation OGs (green bar) and 12-species-new-annotation OGs (purple bar) at the gene level. Hatched boxes label the fraction of mispredicted genes of 34-species- and 12-species-old-annotation datasets that do not exist in Ensembl v60 genome annotations, indicating the high number of errors due to old genome annotations. **C:** Comparison of public eggNOG (yellow bar), 12-species-old-annotation OGs (green bar) and 12-species-new-annotation OGs (purple bar) at the group level. Notice that the 12-species datasets (either with old or new annotation) always introduce a larger number of fission events than the 34-species OGs, highlighting again the importance of species coverage.

Biological complexity is unlikely to be the primary source of errors in automated predicted OGs, as there are single-copy, slow-evolving, or single-domain protein families, which are not assigned correctly by several prediction methods. By investigating these families, we identified two additional technical factors that influence orthology assignment: genome annotation and species coverage. To quantify the impact of these, we used the method in our own hands, eggNOG, as we could apply it to different species sets (Fig. 4, Table S3 of Supporting Information) and genome annotation versions (Fig. 4, Table S4 of Supporting Information).

To measure the impact of species coverage, we prepared new OGs from only the 12 reference species, but kept the same genome annotation version (Ensembl v46) that the public eggNOG v2 uses. The 12-species-Ensembl46 OGs were compared to the RefOGs as well as the 34-species-Ensembl46 OGs (referred to as eggNOG in Fig. 2). In the 12-species-Ensembl46 OGs, a larger number of genes are missing compared to the 34-species OGs (eggNOG_v2) (Fig. 4B), implying that 30% of the missing genes in this dataset are due to the change in species coverage. It seems that sequences of the 34 species facilitate correct clustering, presumably, by breaking long branches so that faster evolving genes can be connected (Fig. 4A). For mammals, fish and insects, which contain more representatives in 34-species OGs, we identified fewer missing genes in the 34-species OGs than the 12-species OGs. On the other hand, *C. elegans* and *C. intestinalis*, which are separated by long branches from their nearest phylogenetic neighbors in both datasets, are not influenced as the sequence similarity for ortholog detection remains limited (Fig. 4A). While 34-species perform better than 12-species in terms of missing genes, they contain more erroneously assigned genes. A large fraction of erroneously assigned genes is due to inclusion of low-quality genomes, i.e. Tetraodon in Ensembl v60 contains almost 5,000 gene predictions less than the same genome in Ensembl v46. In summary, the total number of mispredicted genes is higher for the 12-species OG (Figs. 4A and C), indicating that the more genomes and in particular those at the right evolutionary distance, increase the quality of the OGs.

## Number of errors inflates because of inaccuracies in genome annotation

The quality of the genome annotation of a species included in a genomic or phylogenetic study has been reported to affect the results of the study 42. All resources in this study rely on Ensembl 43 genome annotations for all 12 species, but the annotation status is considerably different from version to version. While eggNOG uses Ensembl v46 (the oldest among the compared resources) OrthoDB uses Ensembl v59, thus it is the most updated and closest to the RefOG annotation, for which Ensembl v60 was used. By tracing the identifiers of the mispredicted genes through Ensembl history, we discovered that 7% of the missing genes of eggNOG only exist in the latest versions of Ensembl (v54 to v60) (Fig. 4B). Genomes like human, zebrafish and puffer fish, which were updated after Ensembl v46, contribute significantly to the pool of missing genes. Likewise, only 58% of the erroneously assigned genes of eggNOG map to Ensembl v60, while 40% of them have been retracted and 2% have been characterized as pseudogenes. Taken together, almost half of all errors result from genome annotation artifacts, which is thus a major factor to consider. To directly test the effect of the genome annotation and separate the impact of species coverage from this analysis, we clustered the proteins of the 12 reference species based on the Ensembl v60 gene annotations. The impact of genome annotation is elucidated by comparing the number of errors between the 12-species-Ensembl60 OGs with the 12-species-Ensembl46 OGs. Comparing the overall number of mispredicted genes, at the gene level, the 12-species-Ensembl60 OGs perform better than the 12-species-Ensembl46 OGs (Fig. 4B). We found 45% fewer erroneously assigned genes (149 vs. 271) in the 12-species-new-annotation OGs compared to the 12-species-old-annotation OGs. Again, a large fraction or erroneously assigned genes of the latter dataset (33%) do not exist in Ensembl v60 (Table S4 of Supporting Information). However, the number of missing genes is similar between the two datasets and higher compared to the 34-species OGs, indicating, once again, the impact of species coverage. The fact that ∼40% of the mispredicted genes in eggNOG OGs would have been avoided by using an updated version of genome annotations, highlights the importance of frequent updates and points to the sensitivity of genome annotations.

## A transparent benchmark set made publicly available

To facilitate the access to the curated benchmark families, we have created a web interface through which details on the 70 RefOGs can be retrieved. In addition, alignments, protein sequences, phylogenetic trees and HMM of each RefOG can be downloaded and used for future analyses of the 70 bilaterian OGs. The data are available under the Creative Commons Attribution 3.0 License at: http://eggnog.embl.de/orthobench.

## Conclusions

The quality assessment introduced here is independent of functional associations and, instead, directly approaches the phylogenetic foundations of OGs. The benchmark set was applied to five commonly used databases and revealed the impact of several biological and technical factors that challenge orthology prediction. All studied repositories predict only a fraction of RefOGs accurately and thus indicate that there is considerable room for improvement for all orthology assignment methods. Although it is impossible to completely quantify the individual factors that contribute to the errors of each method due to the diversity of the methodologies, hidden correlations, and confounding variables, the 70 RefOGs reveal biological and technical limitations that affect each method significantly. For example, domain complexity is significantly correlated with an increased accumulation of erroneously assigned genes in all databases. Our results also illustrate that all the tested algorithms need to be improved to be able to handle the “complex” families (duplication/losses, complex domain architectures). Of the RefOGs, 36% are not accurately predicted by any tested databases, revealing “global” limitations of orthology predictions that are associated with the factors we outlined here. There are also RefOGs that only some of the databases mispredict, and, thus, hint at database-specific improvements, i.e. several operational differences, such as the delineation of hierarchical groups, and the usage of (as close as possible) outgroups affect the accuracy of predicted OGs.

However, the most striking outcome of this study is that technical factors, such as genome quality followed by the phylogenetic coverage of the compared species seem to be the most limiting factors, causing up to 40% of the errors observed. The last observation suggests that frequent updates of the databases are necessary. Although we only tested bilaterian OGs in this study, we realize the importance of the expansion to other taxonomic groups, and have therefore provided sequences, alignments, HMM profiles, and trees of the RefOGs publicly at http://eggnog.embl.de/orthobench for further curation in other species. As this benchmark set proved valuable for assessing the quality of predicted OGs in metazoans, we believe that an analogous dataset covering the entire tree of life and capturing additional challenges more prominent in prokaryotes, such as horizontal gene transfer, should be the next step in guiding orthology prediction.

## Abbreviations:

COG: clusters of orthologous group
EGF: epidermal growth factor
HMM: hidden Markov models
LCA: last common ancestor
MSA: multiple sequence alignment
OG: orthologous group
RefOG: reference orthologous groups
VWC: von Willebrand factor type C
VWD: von Willebrand factor type D
