# Supplementary material for: Orthology prediction methods: A quality assessment using curated protein families
Source: Bioessays. 2011 Oct;33(10):769–80. doi: 10.1002/bies.201100062 (PMC3193375; doi:10.1002/bies.201100062)

Supplementary Information

Quality assessment of orthology prediction methods using curated protein families

Kalliopi Trachana, Tomas Larsson, Sean Powell, Wei-Hua Chen, Tobias Doerks, Jean Muller, Peer Bork

Material and Methods

- Selection of reference families
- Building the Reference Orthologous Groups (RefOGs)
- Mapping of RefOGs to the five databases

Figure S1: The effect of the 35 challenging families on the performance of the methods

Figure S2: The impact of biological complexity in orthology assignment at the group-level (fusions/fissions).

Figure S3: The effect of repeated domains on orthology assignment

Figure S4: The quality of MSA as a proxy for accurate orthology prediction

Table S1: Presentation of the 70 families that consist this benchmark set (separate xls file)

Table S2: Evaluation of the 5 databases using RefOGs (separate xls file)

Table S3: Effect of the species coverage (separate xls file)

Table S4: Effect of the genome annotation (separate xls file)

Table S5: Correlation and p-values of error distribution and error sources

Materials and Methods

Selection of reference families

70 Orthologous Groups (OGs) were selected from the second version of the eggNOG database (*Muller et al, 2010*), the majority of which were originally build for the COG database (*Tatusov et al, 1997*), to form a benchmark set of orthology prediction. Previous studies have reported certain biological (e.g. multi-gene families) or technical aspects (e.g. quality of MSA) that cause problems in assignment of orthologous groups (*Tatusov et al, 1997; Koonin EV, 2005; Gabaldon T, 2008).* 40 out of the 70 selected families were classified under a specific category of biological or technical challenge, while 30 OGs were selected randomly (Table S1). The trouble-making categories that are covered by our benchmark set are the following:

1. Multiple Sequence Alignment Quality: To select OGs with different alignment quality, we built multiple sequence alignments (MSA) of every OGs either at the universal (similar to COG) or eukaryotic-specific (similar to KOG) level on eggNOG database. The MSA were computed using the AQUA protocol (*Muller et al, 2009*) setup to use MUSCLE (v3.7) (*Edgar RC, 2004*) and RASCAL (v1.34) (*Thompson et al 2003*). AQUA makes use of the NORMD program (*Thompson et al. 2001*) to assess the quality of each individual MSA by comparing norMD scores and selecting the one with the highest score. The norMD score gives information about the general quality of the alignment, a norMD >0.6 indicates a reliable MSA, (*Thompson et al., 2003*). Looking at the distribution of the norMD score for all OGs, one can observe first, that the OGs dataset does contain the full spectrum from fast to slow evolving gene families and second, that the vast majority of the gene families have a reliably aligned MSA (i.e. norMD>0.6). 10 families were selected under this category; 8 of them represent families with low quality MSA (norMD<0.6), while 3 of them score a high quality MSA (norMD>2).

2. Speed of evolution: The multiple sequence alignments were also used to define the speed of evolution. To classify eggNOG OGs based on their evolutionary pace, we computed the mean percent identity for each of them. The mean percent identity (described as the "FamID" in *Muller et al, 2006*) is calculated as the mean pairwise percent identity of each sequence against each other within a given MSA. Positions in the alignment corresponding to gaps within the MSA were excluded from the calculation.

$$FamID = 2\frac{\sum_{1\le i<j\le n} ID_{S_i,S_j}}{n(n-1)}$$

where:

n = total number of sequence tested, S_i and S_j are the *i*th and *j*th sequence,

IDs_i,s_j = pairwise percent identity between the *i*th and *j*th sequence, excluding gap regions.

Only 2 of the 10 selected families for this category are slow evolving families, while the rest eight are characterized as fast-evolving families (MeanID<0.45) (Table S1).

3. Low complexity/repeats: Intrinsic features like low complexity, coiled coil and other variable repeated elements or repeated modules can affect the building of orthologous groups. 10 families were taken from this category.

4. Domain complexity/Domain shuffling: The complexity of the domain architecture of different protein families can lead to miss-assignment of orthologs. The vast majority of proteins consist of single or a few (2-3) domains; however, we collected 4 OGs that have been previously reported to hamper orthology prediction either due to the complex architecture within the protein (contain more than 4 domains) or due to the high variety of domain architecture among the members of an OG.

5. Multigene families: Of all above problem, the most-acknowledged one that affects all three domains of life is the multi-gene families and the detection of paralogy. 6 large OGs that contain several paralogs and orthologs were chosen to address this issue.

Building the Reference Orthologous Groups (RefOGs)

Starting with COG/KOGs (Table S1) as "homology seeds" we manually recovered orthologous groups for 12 bilaterian species that are referred to as Reference Orthologous Groups (RefOGs). Initially, we mapped the "homology seed" identifiers (Ensembl v46) to Ensembl v60 via Ensembl History. BLAST (*Altschul et al, 1990*) searches were performed in the 12 reference genomes and four outgroup species (*Monosiga brevicollis*, *Trichoplax adherens*, *Nematostella vectensis* and *Hydra magnipapillata*) using query sequences from well-annotated genomes (e.g. human, zebrafish and fly). The homologous sequences were aligned by MUSCLE (*Edgar, 2004*) and the alignments were used to build NJ trees with Clustal X (Larkin et al, 2007). Large groups were resolved by the presence of ortholog(s) in the outgroup(s) (Figure 2). However, in several cases no clear outgroup was found hampering the resolution on the bilaterian level. For these families, RefOGs were defined based on i) the domain content using SMART database (*Letunic et al., 2009*), ii) manual inspection of the alignments and iii) previous published descriptions of the families. After the initial curation of the families, all sequences determined to be members of the bilaterian RefOGs were aligned using MUSCLE (*Edgar, 2004*). Alignments were manually cut based on the first and last well aligned columns according to GBLOCKS (*Castresana J, 2000*) with the following parameters: (Minimum Length Of A Block: 10, Allowed Gap Positions: With Half, Use Similarity Matrices: Yes). This was made in order to remove highly divergent N- and C-terminal parts of each alignment where misalignment is assumed to be common. Alignments were further manually cleaned to remove large parts where all sequences but one had gaps or short sequences that did not align within a conserved "block". Based on the refined alignments, Hidden Markov Models (HMM) were built using the HMMER3 package (*Eddy SR, 2009*). At a second refinement step, the HMM models were used to identify related sequences that were left out from the 16 aforementioned genome. We did not define a global cut-off for sequence recruitment instead we treat each family uniquely by adding sequences with bit score within the range of bitscores of already known members. After the addition of those sequences phylogenetic trees were calculated using PhyML version 3.0 (*Guindon et al, 2010*) with the following settings: 100 bootstrap replicates, optimization of tree topology, branch lengths and rate parameters, 4 substitution rate categories and the NNI topology search option. RefOG identifiers, alignments, HMM models and trees are available on www.eggnog.embl.de/orthobench.

Mapping of RefOGs to the five databases

Five orthology prediction methods were benchmarked against the RefOGs: TreeFam (release 7.0), eggNOG (v2.0), orthoDB (customized orthologous groups for the 12 reference species), orthoMCL (v4.0) and OMA (release 3.0). Generally, we downloaded and benchmarked the latest version of each database (October 2010). TreeFam resolve the evolutionary relationships of big homologous families through tree reconciliation, thus we had to score each RefOG against the reconciled tree of the respective homologous family on the bilaterian level. TreeFam provides both curated and automatically predicted orthologous groups, we used the second category for our analysis. eggNOG generates OGs for different taxonomic levels, thus, in the current comparison we used OGs generated by bilaterian species only (called meNOGs). eggNOG and OrthoDB use a similar clustering procedure based on triangulars of best hits; OrthoMCL identifies OGs using Markov clustering and OMA applies its unique algorithm, which does not allow paralogs within OGs.

The RefOGs are built using the genome annotations of Ensembl_v60. However, all five repositories predicted OGs based on older Ensembl versions. For each RefOG sequence we track its identifiers to older Ensembl versions via Ensembl History (i.e. ENSTNIG00000002616 (annotated as RPL11 Ensembl_v60) mapped to GSTENG00003639001 in to Ensembl_v46). There are certain cases, where this automated procedure doesn't work, i.e. one protein of Ensembl_v60 maps to multiple identifiers in older Ensembl releases or genome assemblies predict a new gene locus (e.g. ENSDARP00000103772 (prok1) - a predicted locus after Ensembl v54- is identified as a missing ortholog in eggNOG, orthoMCL and TreeFam databases that use older releases of Ensembl).

References

1. **Tatusov RL, Koonin EV, Lipman DJ.** 1997, A genomic perspective on protein families. *Science* **278:** 631–637.

2. **Ruan J, Li H, Chen Z, Coghlan A,** *et al.* 2008. TreeFam. 2008 Update. *Nucleic Acids Res.* **36**(Database issue): D735-40.

3. **Muller J, Szklarczyk D, Julien P, Letunic I,** et al. 2010. eggNOG v2.0. extending the evolutionary genealogy of genes with enhanced non-supervised orthologous groups, species and functional annotations. *Nucleic Acids Research* **38**(Database issue): D190-195.

4. **Waterhouse RM, Zdobnov EM, Tegenfeldt F, Li J, Kriventseva EV.** 2011. OrthoDB. the hierarchical catalog of eukaryotic orthologs in 2011. *Nucleic Acids Res.* **39**(Database issue): D283-288.

5. **Chen F, Mackey AJ, Stoeckert CJ Jr, Roos DS**. 2006. OrthoMCL-DB. querying a comprehensive multi-species collection of ortholog groups. *Nucleic Acids Res.* **34**(Database issue): D363-368.

6. **Altenhoff AM, Schneider A, Gonnet GH, Dessimoz C**. 2011. OMA 2011. orthology inference among 1000 complete genomes. *Nucleic Acids Res.* **39**(Database issue): D289-294.

7. **Muller J, Oma Y, Vallar L, Friederich E**, *et al.* 2005. Sequence and Comparative Genomic Analysis of Actin-related Proteins. *Molecular Biology Cell* **16**(12): 5736-48.

8. **Letunic I, Doerks T, Bork P**. 2009. SMART 6. recent updates and new developments. *Nucleic Acids Res.* **37**(Database issue): D229-232.

9. **Thompson JD, Plewniak F, Ripp R, Thierry JC**, et al. 2001. Towards a reliable objective function for multiple sequence alignments. *J. Mol. Biol.* **314**: 937-951.

10. **Thompson JD, Thierry JC, Poch O.** 2003. RASCAL. rapid scanning and correction of multiple sequence alignments. *Bioinformatics* **19**.1155-1161.

11. **Muller J, Creevey CJ, Thompson JD, Arendt D**, *et al.* 2009. AQUA. Automated quality improvement for multiple sequence alignments. *Bioinformatics* **26**(2): 263-5.

12. **Flicek P, Amode MR, Barrell D, Beal K**, *et al.* 2011. Ensembl 2011. *Nucleic Acids Res.* **39**(Database issue): D800-806.

13. **Edgar RC.** 2004. MUSCLE. multiple sequence alignment with high accuracy and high throughput. *Nucleic Acids Res*. **32**(5): 1792-7

14. **Sorek R, Zhu Y, Creevey CJ, Francino MP,** *et al.* 2007. Genome-wide experimental determination of barriers to horizontal gene transfer. *Science.* **318**(5855): 1449-52

15. **Garcia-Vallve S, Guzman E, Montero MA, Romeu A**. 2003. HGT-DB. a database of putative horizontally transferred genes in prokaryotic complete genomes. *Nucleic Acids Research* **31**: *187-189.*

16. **Altschul SF, Gish W, Miller W, Myers EW,** et al. 1990. Basic local alignment search tool. *J Mol Biol.* **215**(3): 403-10.

17. **Larkin MA, Blackshields G, Brown NP, Chenna R,** *et al.* 2007. Clustal W and Clustal X version 2.0. *Bioinformatics* **23**(21): 2947-8.

18. **Castresana J**. 2000. Selection of conserved blocks from multiple alignments for their use in phylogenetic analysis. *Mol. Biol. Evol.* **17**: 540-552.

19. **Eddy SR**. 2009. A new generation of homology search tools based on probabilistic inference. *Genome Inform.* **23**(1): 205-11.

20. **Guindon S, Dufayard JF, Lefort V, Anisimova M**, *et al.* 2010. New algorithms and methods to estimate maximum-likelihood phylogenies. assessing the performance of PhyML 3.0. *Syst Biol*. **59**(3): 307-21.

Figure Legends

Figure S1: A benchmark set that highlights the challenges of orthology assignment. Using the manually curated RefOGs, we evaluated the performance of five databases. Among the 70 families, there are 35 families that illustrate challenges of orthology prediction. Green bar illustrates the performance of the databases for those 35 families, while the purple bar shows the performance of the databases for all the benchmark set. The upper panels illustrate the accurately predicted RefOGs at two different levels (gene- and group-level). The low panels show the % effected RefOGs by 4 different errors: erroneously assigned genes - missing genes (left) and fusion - fissions (right). The larger green bars on the lower panels illustrate the higher number of errors that accumulate the 35 complicated families.

Figure S2: The impact of biological complexity in orthology assignment at the group-level (fusions/fissions). *(A) The impact of family size (paralogy);* The RefOGs were separated into (i) small (contain less than 14 members), (ii) medium (contain more than 14 members, but less than 40) and (iii) large (contain more than 40 genes). For the graph-based methods (eggNOG, OrthoDB, OrthoMCL and OMA), we observe that they split larger RefOGs into more orthologous groups than the smaller ones. *(B) Speed of evolution;* The RefOGs were classified based on the MeanID score (described as the “FamID” in *Muller et al, 2006*), an evolutionary rate score derived from the multiple sequence alignment of each family. There are (i) slow-evolving (MeanID>0.7), (ii) medium-evolving (MeanID lower than 0.7, but larger than 0.5) and (iii) fast-evolving (MeanID<0.5) RefOGs. Similarly to the previous observation, as biological complexity increases (slow to fast-evolving families), we count more fission events for the graph-based methods. *(C) Domain architecture complexity;* each RefOG is associated with the average number of domains, which is equal to the sum of predicted domains of the members of one RefOG divided by the family size. Again, there are 3 levels of complexity, starting from (i) none or 1 domain on average to (ii) 2-4 to (iii) more than 4. By classifying RefOGs based on their domain complexity we can see a more diverse pattern; TreeFam seems to have a large number of fusion events on the most difficult category, while OrthoMCL seems to have a uniform distribution of fissions across all three categories. Significant correlations (Table S5) between the distribution of missing/erroneously assigned genes and the tested factor is indicated with an asteric.

Figure S3: Repeated domains affect the orthology assignment. We used SMART database to identify the number of domains for each protein of our benchmark dataset. 24 out of the 70 RefOGs contain proteins with repeated domains (Table S5). We observed that the percentage of RefOGs that failed to be predicted accurately is higher for these 24 families than the rest indicating that repeated domains have an impact in orthology assignment.

Figure S4: The quality of MSA as a proxy for accurate orthology prediction. (A) We classified the families based on their norMD score (*Thompson et al, 2001*) into (i) high quality alignment (norMD>0.6) and (ii) low quality alignment. We observed that all graph-based methods tend to have more fissions when the alignment quality is low. For TreeFam, on the other hand, low quality of alignment was correlated with fusions. (B) Effect of sequence length variation; the RefOGs were divided into three different categories (low, medium and high deviation) based on the sequence length variability of included orthologs. We can see that RefOGs with variable-size members accumulate the higher fraction of fusion and fission events. Significant correlations (Table S5) between the distribution of missing/erroneously assigned genes and the tested factor is indicated with bold letters. Significant correlations (Table S5) between the distribution of missing/erroneously assigned genes and the tested factor is indicated with an asteric.

Figure S1.

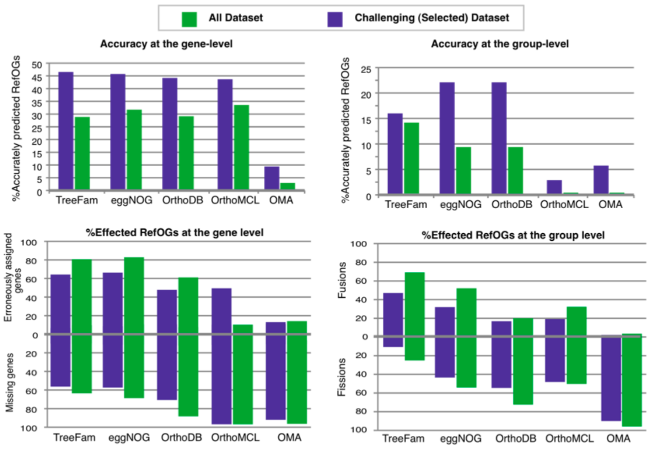

Figure S2.

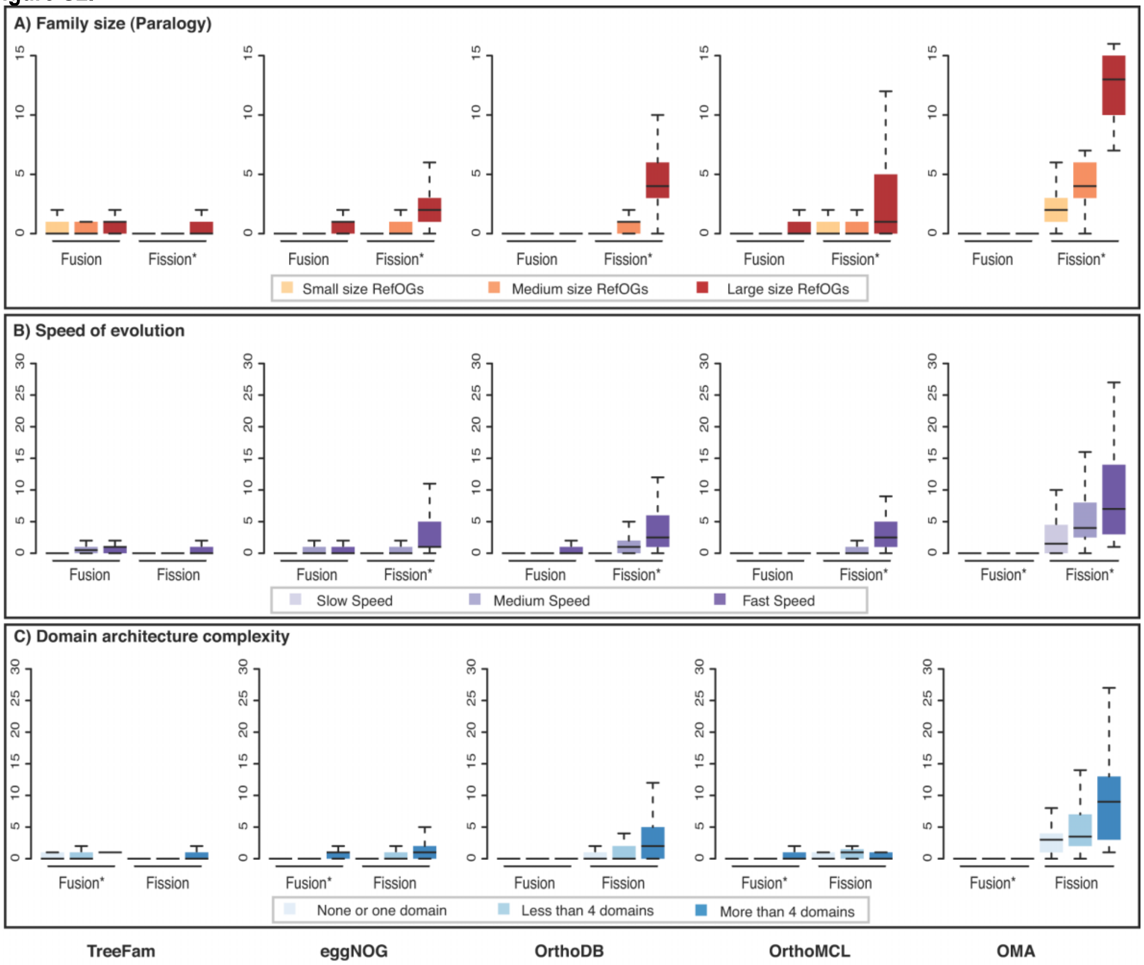

Figure S3.

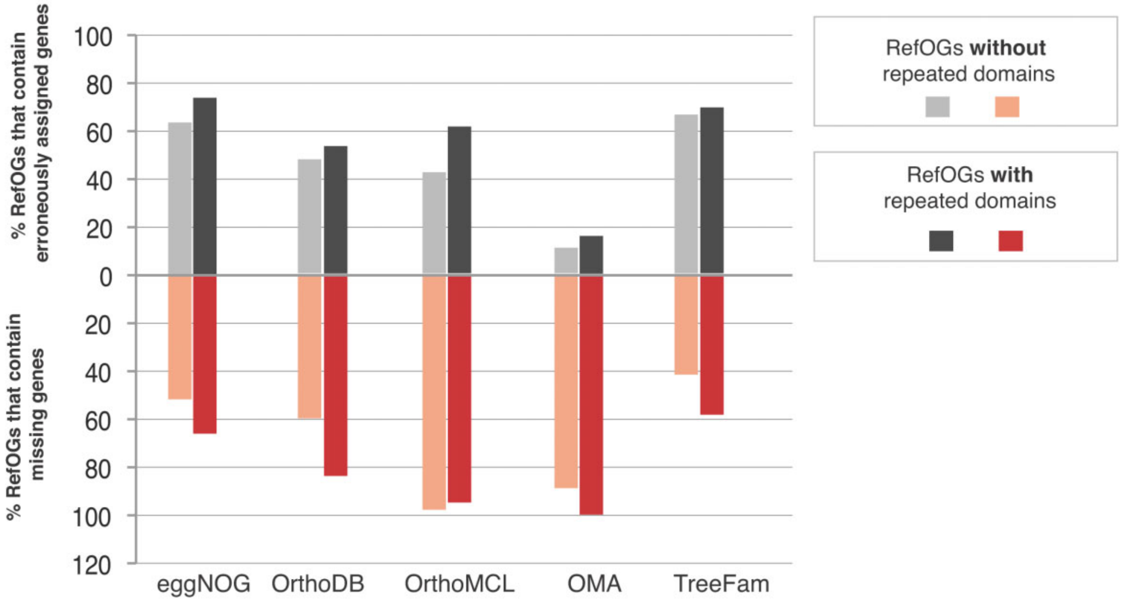

Figure S4.

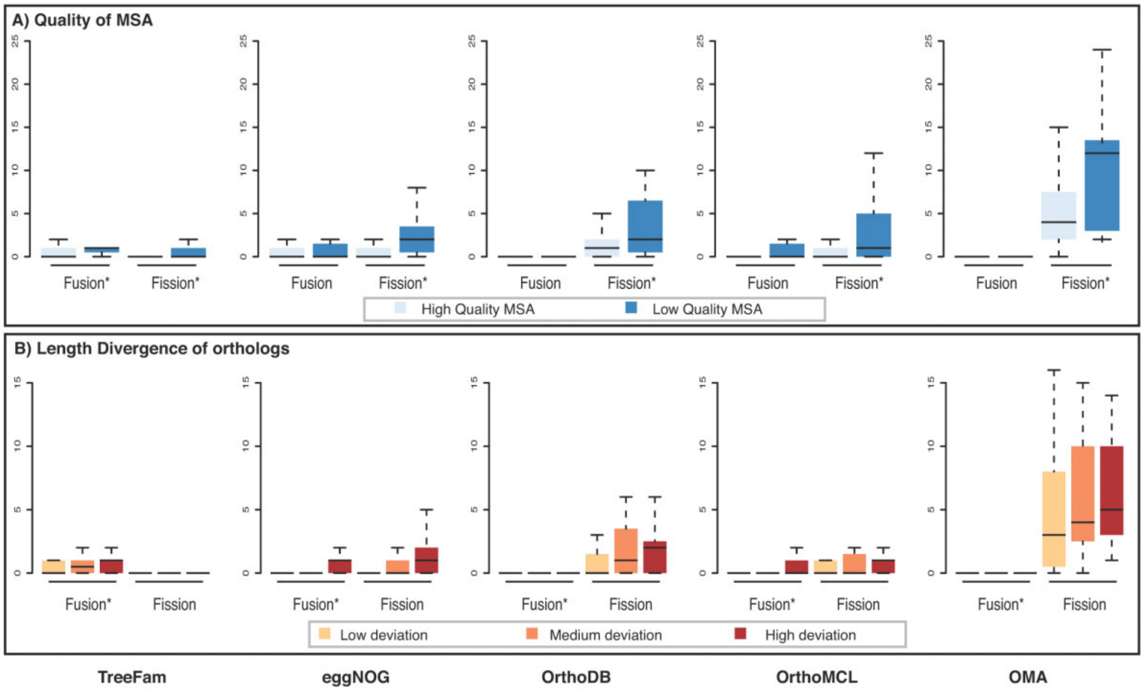

Supplement: Supplementary file 1 [file bies0033-0769-SD1.pdf]
